# Olfactory impairment in men and mice related to aging and amyloid-induced pathology

**DOI:** 10.1007/s00424-021-02527-0

**Published:** 2021-02-20

**Authors:** Wen-Yu Tzeng, Katherine Figarella, Olga Garaschuk

**Affiliations:** Institute of Physiology, Department of Neurophysiology, Eberhard Karls University of Tübingen, Keplerstr. 15, 72074 Tübingen, Germany

**Keywords:** Olfaction, Olfactory memory, Odor identification, Normal aging, Alzheimer’s disease, Amyloid plaques

## Abstract

Olfaction, or the sense of smell, is one of the most ancient senses in men and mice, important for a large variety of innate and acquired behaviors. Clinical data reveal an early impairment of olfaction during normal aging and in the course of neurodegenerative diseases, but the underlying cellular/molecular mechanisms remain obscure. In the current review, we compare different aspects of the aging- and Alzheimer’s disease related impairment of olfaction in men and mice, aiming at the identification of common morbidities and biomarkers, which can be analyzed in detail in the appropriate mouse models. We also identify common, often interdependent (patho)physiological pathways, including but not limited to extracellular amyloid depositions, neuroinflammation, ɛ4 allele of the apolipoprotein E, CNS insulin resistance, and the impairment of adult neurogenesis, to be targeted by basic and clinical research.

## Introduction

The sense of smell is one of the most ancient senses [138]. In mammals, this sense is important for (i) food localization, (ii) investigation of and navigation through the environment, (iii) social interaction and courtship behaviors, (iv) food-preference determination, (v) regulation of the appetite, and (vi) emotional contagion [55, 138, 149]. Accordingly, the largest part of the mammalian genome is occupied by genes encoding the olfactory receptors (approximately 900 genes in mice and 1500 genes in humans) [170]. Although in humans the sense of smell is less important for everyday life as, for example, hearing or vision, it represents an important constituent of gustatory and visual perception, psychosexual functions, aggression and fear learning, attraction and aversion behaviors, gastrointestinal functions and, via close connections with the limbic system, strongly impacts human emotions and memories [28, 54, 63, 77, 90, 134, 138, 151].

Interestingly, the sense of smell is also an early indicator of many viral (COVID-19 being the latest example) and bacterial infections [36, 98, 127, 157] as well as neurodegenerative diseases [46, 101]. Olfactory tests are, for example, routinely used in the clinic during the early diagnostics of Alzheimer’s (AD) and Parkinson’s (PD) diseases as well as mild cognitive impairment (MCI) [58, 80, 105, 172]. However, our understanding of the olfactory dysfunction in aging is still very finite. Moreover, it is confounded by the difficulty to separate normal elderly control (NEC) subjects from prodromal PD/MCI/AD cases, because olfactory disturbances are among the very first biomarkers of emerging neurodegenerative pathologies. Currently, most available studies do not include longitudinal follow-up, which would be mandatory for disentangling the olfactory dysfunction caused by aging per se from the dysfunction caused by the beginning age-related pathologies.

Here we review the current knowledge about the aging-related olfactory dysfunction in humans and mice. We discuss the similarities and the differences to find out to which extent the mouse models can help to understand the cellular and molecular mechanisms of olfactory impairment during normal aging and in the course of neurodegenerative diseases.

## Olfactory impairment in the elderly

The olfactory system is capable of odor detection, odor discrimination, and olfactory memory and employs these capabilities in a wide range of behaviors (e.g., food localization, environmental investigation, or social interaction). The underlying computations are performed by different brain circuits, and their function is assessed experimentally by different tasks using various compositions and concentrations of odorants [139]. One common olfactory test is the odor detection threshold test. Subjects are asked to smell the different concentrations of an odor to identify the lowest concentration they can discern. The odor identification test, measuring the ability to identify and distinguish different odorants, is also widely used. In addition to the sense of smell, this test requires the ability related to semantic memory. Indeed, the specific odor memory has to be acquired during the initial odor presentation and correctly recalled with a verbal label upon the subsequent (test) presentation of the same odor [131, 140]. Therefore, it requires not only the ability of odor detection but also an intact olfactory memory for specific odors. In contrast to odor identification, *odor discrimination* is an ability to distinguish two or more odors against the background. In different tasks, the subject is required to distinguish similar or dissimilar odors. In addition to odor discrimination tasks, the tests also assess the ability to distinguish different concentrations of the same odor. Interestingly, discrimination of odor quality activates brain networks, which are more related to the cognitive function than the networks, activated when discriminating the odor intensity [139], suggesting that these two functions are mediated by different neural circuits. This suggestion is further supported by the patient data showing that bilateral removal of the medial temporal lobe impairs the discrimination of the odor quality but not the odor intensity [53].

Olfactory impairment refers to a decrease in the ability of odor detection, odor discrimination, and olfactory cognition. It represents a common characteristic of advanced age, with hyposmia (i.e., the reduced ability to smell and to detect odors) increasing with age both in men and women [111, 141–143]. How the increasing age impacts the abovementioned abilities of odor detection, odor discrimination, and olfactory cognition is reviewed below.

### Odor detection

Schubert et al. [144] measured odor detection thresholds in men and women of advanced age (68–99 years old). They found that older participants (≥ 85 years of age) were significantly more likely to have a worse detection threshold than younger (68–74 years old) participants. There was, however, no difference in odor detection between men and women. These results are consistent with the findings of another group [128], who observed that participants older than 60 years had significantly higher odor detection thresholds compared to younger (≤ 45 years old) participants. Moreover, in the younger age group, the odor detection thresholds remained stable between 20 and 45 years of age, whereas in the older age group, there was an age-associated decline in the odor sensitivity.

These findings clearly suggest that the odor detection threshold increases and the olfactory detection ability declines with increasing age.

### Odor identification

**Fig. 1:**
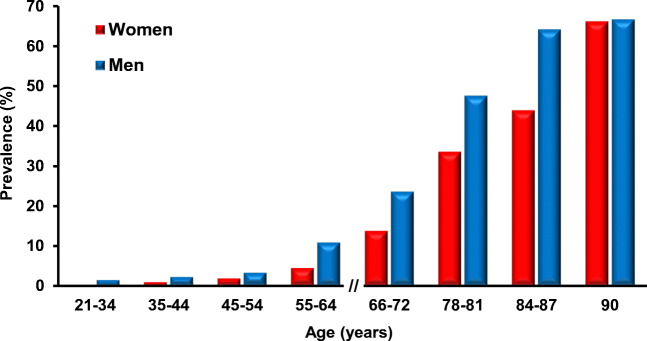
Prevalence of olfactory impairment in participants of different age and sex. Figure is adapted from refs. [143, 145]

Odor identification requires an ability to sense and to identify an odor [84]. This ability, especially its cognitive aspect, might potentially be influenced by many factors including age, gender, and personality. Previous studies have found a decline in the ability of odor identification with age. Especially, individuals over 80 years of age show a high prevalence of olfactory impairment [48, 111, 143]. Murphy et al. [111] used the San Diego Odor Identification Test (SDOIT) utilizing natural odors to show that 80–97-year-old people are impaired in odor identification abilities. In 2003, Choudhury et al. used an odor memory test, which requires the ability of odor identification and the ability to evaluate the odor identification ability in 10–68-year-old subjects of both genders. The results showed that the odor identification ability and the ability to correctly assess the odor intensity starts to decline at the age of 20 years in men but not until the age of 40 years in women [29]. Hori et al. applied an odor identification ability test in healthy subjects of different ages: young adults (20–43 years old), middle-aged adults (45–69 years old), and old adults (70–89 years old). They found that the group of old adults exhibited lower odor identification ability compared to young adult and middle-aged adult groups [68]. Other study assessed the olfactory function in 21- to 84-year-old participants using the SDOIT and found that the prevalence of olfactory impairment is increasing with age from 0.6% in young adult (< 35 years old) participants to 13.9% in elderly (≥ 65 years old) population [143]. When compared by decades of life, the authors observed a continuous increase in the prevalence of olfactory impairment. There were also profound gender differences with women performing better than men in all age groups and men’s scores declining significantly faster with age (Fig. 1).

In addition to these single time point measurements, the same group has conducted a longitudinal population-based study of sensory loss during aging [142]. This study started at the age of 53–59 years and the prevalence of the olfactory impairment was 3.7% and 4.6% among 53–59-year-old women and men, respectively. As the population aged, the prevalence of olfactory impairment increased steadily amounting to 19.3% and 24.8% in 70–79-year-old women and men, respectively. In the 80–97-year-old population, the respective numbers for women and men were 52.9% and 31.6%. In this study, one out of every eight older adults (beginning at age of 53–59 years) acquired an olfactory impairment over 5 years. While women again performed equally well or better than men in all age groups, the rate of decline in women’s scores caught up to men at the oldest (80–97 years old) age group [142, 147].

Taken together, these data convincingly show that the odor identification ability declines with increasing age. Interestingly, women have better odor identification ability than men in all age groups. At the end of life, however, women and men reach a comparable level of decline. Furthermore, these data clearly document the profound nature of the aging-associated impairment of olfaction, negatively impacting the quality of an individual’s life.

### Olfactory memory

Olfactory memory refers to odor-evoked memory and the recognition of the learned or familiar odors [163] and is often used to test the cognitive function in the elderly. The Sniffin’ Sticks test (Burghardt®, Wedel, Germany), originally established by Hummel et al., is used to assess olfactory function and contains three indexes: odor threshold, odor discrimination, and odor identification [70, 135]. It can also be used as the Sniffin’ TOM (test of odor memory) test, to test the olfactory memory. The olfactory memory acquisition and expression requires the ability to detect an odor, to acquire an intact olfactory memory for specific odors, and to retrieve this odor label from semantic memory upon retest. In 2015, Croy et al. applied a modified the Sniffin’ Sticks test to encode odor memory in 3 age groups (young adults with the mean age of 24.5 years, middle-aged adults with the mean age of 48.6 years, and old adults with the mean age of 69.8 years) and then conducted a yes-no recognition test and odor identification test. After 17 days, they retested the ability to identify all odors using the Sniffin’ TOM test. They found that the performance in the Sniffin’ TOM test in these three groups declined with increasing age [33]. In 2016, Larsson et al. assessed olfactory memory in 60- to 100-year-old participants by the Sniffin’ Sticks test and found an age-related decline in olfactory memory performance [85]. Recently, Seubert et al. applied a modified Sniffin’ Sticks identification test in a healthy population (mean: 69.73 ± 8.76) and found that aging was significantly correlated with episodic (odor recognition memory) and semantic (odor naming) [56] olfactory memory performance [146]. Furthermore, they found positive associations between semantic olfactory memory performance and regional gray matter volume in the amygdala, posterior piriform cortex, and anterior hippocampus [146]. The piriform (olfactory) cortex projects to the amygdala and hippocampus and these connections are involved in emotion and memory [107], implying that olfactory function is correlated with cognitive function in the elderly. Interestingly, even though the elderly participants showed a decline in olfactory cognitive abilities, non-olfactory cognitive abilities were less impaired [71], suggesting that olfactory dysfunction is an early indicator of cognitive decline.

### Functional connectivity in the olfactory network

Cognitive and olfactory functions decline in parallel during normal aging [21, 108, 158]. Moreover, elderly people with olfactory dysfunction often show poor cognitive abilities [42]. However, the link between the two aging-related disabilities is obscure. One approach is to study the aging of functional connectivity in the olfactory network by resting-state functional magnetic resonance imaging (fMRI) [154]. Karunanayaka’s lab reported that the resting-state olfactory network shows extended functional connectivity to the thalamus, medial prefrontal cortex, caudate nucleus, nucleus accumbens, parahippocampal gyrus, and hippocampus [154]. Furthermore, they investigated the effect of aging (20–61-year-old subjects) on the resting-state olfactory network and found that functional connectivity between the olfactory network and parahippocampal gyrus was negatively correlated with age [74]. A recent study [166] investigated the effect of aging on global functional connectivity by resting-state fMRI in young (20–39 years old), middle-age (40–59 years old), and elderly (age ≥ 60 years old) participants. The data revealed a negative correlation between aging and global functional connectivity, including the areas, which relate to the olfactory network and cognitive function (e.g., olfactory, orbital, medial prefrontal cortex, and amygdala).

Taken together, these data suggest that in part, the age-related impairment of the olfactory and cognitive function may be caused by the decline of functional connectivity within the olfactory network.

## Early olfactory impairment in patients with Alzheimer’s disease

AD is a complex age-related neurodegenerative disorder that is characterized by aberrant accumulation of the amyloid β (Aβ) protein and its precipitation in the senile plaques, formation of neurofibrillary tangles (composed of aggregates of microtubule-associated protein tau), cognitive disability, dementia, olfactory dysfunction, and the progressive loss of neuronal structure and function. Cumulative evidence suggests that soluble oligomeric forms of Aβ initiate a vicious cycle leading to the deposition of dense-core plaques, activation of the brain’s immune system, tau misfolding, and assembly of neurofibrillary tangles. Subsequently, these pathologies spread throughout the cortex, resulting in neural network dysfunction, neurodegeneration, and cognitive decline. Amyloid plaques are initially deposited in the neocortex, particularly in medial prefrontal and medial parietal regions, spreading from there to the neighboring cortical areas as well as the hippocampus and eventually infiltrating all cortical areas. Tau aggregates first appear in the entorhinal cortex and hippocampus, propagating to limbic and association areas as the disease progresses [14, 18]. While plaque deposition is also found in the brains of cognitively normal individuals, the tau pathology is more closely related to neuronal loss and clinical symptoms of AD. Tau aggregates are also abundant in all layers of the olfactory bulb, while amyloid plaques are mostly found in the anterior olfactory nucleus [118]. The prevalence of late-onset AD within the world’s population is rising, and the number of patients is expected to double by 2050, causing numerous medical and social burdens [65, 67, 102]. However, the decade-long development of the disease makes the study of underlying mechanisms difficult and points out the urgent need for early disease diagnostics.

Early olfactory dysfunction is a common feature of AD and Parkinson’s disease and has been widely described in the medical literature [58, 80, 105, 172]. In fact, olfactory deficits are observed in 85–95% of AD patients, and therefore can be considered a sensitive marker of AD [50]. AD patients often show impairments in odor detection threshold, odor identification and odor recognition capabilities [125] as well as marked structural and biochemical alterations in the olfactory bulb (OB) and the entorhinal cortex, brain regions important for olfactory function [40, 47, 110]. A reduced volume of the OB has been typically found in AD patients with a higher burden of neurofibrillary tangles and minimal mature amyloid plaques [22, 109, 152], accompanied by the atrophy of the olfactory glomeruli, loci, where olfactory receptor neuron synapse on mitral and tufted cells [22]. Senile plaques have been also found in the olfactory epithelium [83], and neuronal loss has been reported in the whole olfactory system, including the olfactory epithelium, OB, anterior olfactory nucleus, and the higher cortical centers [35, 82, 150]. Bellow, we discuss when during the development of AD pathology and how the main features of the olfactory processing become impaired.

### Odor detection

Studies in AD or mild cognitive impairment patients have revealed deficits in the odor detection threshold. Djordjevic et al. [43], for example, evaluated the detection threshold in male and female AD patients (*n* = 27; 14M/13F), MCI patients (*n* = 51; 25M/26F), and normal elderly control subjects (*n* = 33; 17M/16F) of the similar age (55–88, 59–86, and 63–87 years, respectively). They found that AD and MCI patients had significantly higher odor detection thresholds and thus lower olfactory sensitivity than NEC subjects. Besides, they noticed that AD patients had lower olfactory sensitivity compared to MCI patients. These data suggest the quantitative relationship between the degree of AD pathology and the degree of impairment in olfactory sensitivity. Using the Sniffin’ Sticks Threshold Test, Li et al.[91] have also found the lower performance in odor detection (threshold sensitivity) in the early-stage AD group (age: 75.7 ± 4.1; 4M/6F) compared to the age-matched control group (76.3 ± 3.9; 4M/6F). This difference, however, did not reach the level of statistical significance. A recent study on somewhat younger AD patients (69.5 ± 9.4 years old) also failed to find any difference in the odor threshold sensitivity between AD patients and the age-matched (62.5 ± 6.8 years old) control group [44].

In summary, at a younger age and/or in the early stage of the disease, AD patients seem to exhibit rather normal odor threshold sensitivity. Thus, the impairment of odor threshold sensitivity is probably not the earliest symptom in AD-mediated olfactory system dysfunction. However, it becomes progressively more apparent during the development of the disease. Here and below, one has to keep in mind that olfactory dysfunction is also observed in other aging-related disorders (e.g., PD as well as other synucleinopathies) with decade-long prodromal phases. The latter might remain unrecognized when ascribing the subjects to different study groups.

### Odor identification

Recent studies have demonstrated that AD patients are often impaired in the ability to correctly identify a given odorant [44, 68, 72, 91, 123]. Typically, this disability is much more profound than the problem with the odor detection threshold [125]. The University of Pennsylvania Smell Identification Test (UPSIT) is one of the commonly used tests to assess this capability [49]. Djordjevic et al. [43] used the UPSIT to test the odor identification ability in AD and MCI patients as well as NEC subjects. Compared to NEC subjects, both MCI and AD patients showed gradually decreasing odor identification abilities with significant differences between the three groups (see also ref. [72]). Similar results were also obtained by other groups [44, 68, 91], reporting significantly lower odor identification ability of 60–75-year-old AD patients compared to age-matched controls.

Interestingly, not only AD but also MCI patients and subjects with prodromal or preclinical stages of AD have difficulties with odor identification [123]. This impairment turned out to be more severe in MCI individuals with memory impairment (amnestic MCI) compared to MCI individuals without memory impairment (non-amnestic MCI) [123].

Taken together, the studies document a profound and early-onset impairment in olfactory identification ability and suggest that this impairment is exacerbated by accompanying cognitive impairments.

### Odor discrimination

The process of odor discrimination requires both odor detection and identification and relies on the retrieval and processing of the stored odor information [38]. It, therefore, does depend on the intact working memory as well as episodic memory of odor percepts and thus might turn out to be a better biomarker of AD than the described above odor identification ability, which is also impaired in cognitively normal Parkinson’s patients and aged individuals (see above). Consistently, Djordjevic et al. has found that compared to NEC subjects, both AD and MCI patients show the impaired ability of odor discrimination with significant differences between all three groups [43]. Dhilla Albers et al. confirmed these results and found that worse odor discrimination performance was associated with reduced adjusted hippocampal volume and thinner entorhinal cortices [41]. The odor discrimination measure, however, was also influenced by age, gender, and education. The authors concluded that these multiple associations potentially complicate the interpretation of odor identification and odor discrimination deficits as biomarkers in clinically normal populations. They do, however, possess enough power for early diagnostics of AD pathology [41].

### Olfactory memory

A classic approach to study long-term odor recognition memory is based on a yes-no task, in which two sets of stimuli are presented with minute-to-hour-long intervals. The first set of stimuli allows for odor exploration and memory encoding, whereas the second (test) set of stimuli comprises “known” stimuli from the first set together with novel stimuli. Analyses of the long-term odor recognition memory in patients with mild-to-moderate AD (73.0 ± 11.2 years old) and age-matched healthy subjects (67 ± 12.7 years old) revealed clear memory deficits in the AD group [117]. However, it was also found that patients with mild-to-moderate AD had poor olfactory recognition performance only for unfamiliar odors, suggesting that the olfactory impairment in AD patients is mostly caused by impaired memory acquisition. Using a similar approach Dhilla Albers et al. have tested cognitively normal participants (75 ± 1.01 years old) and possible or probable AD patients (77 ± 2.60 years old) and found that the patients perform at chance level in the olfactory memory test [41]. Consistently, among clinically normal elderly performing well in the olfactory memory test, the individuals with ApoE ε4 allele (a known genetic risk factor for AD) were found significantly less frequently [41].

### Functional connectivity in the olfactory network

The olfactory network, consisting of the primary olfactory cortex, hippocampus, insula, and striatum [73, 154], processes environmental chemical signals, transferring them into odor perception [62, 87]. Impairments of the functional connectivity in the olfactory network have been shown in early-stage AD patients [96, 97], and are also present in patients with MCI [96]. Besides, AD pathology is well-known to involve the hippocampus [15], thus impacting memory formation as well as information transmission and reception [167]. A recent study in age-matched cognitively normal subjects, early and late MCI and AD patients showed that functional connectivity between the olfactory network and hippocampus, calculated using low-frequency fMRI signal fluctuations, became progressively disrupted across disease states, with significant differences between early and late MCI groups. This disruption happens before the atrophy of the hippocampal tissue [96].

Besides the olfactory network, the default mode network is also important for memory function and is vulnerable to aging, amyloid deposition, and AD [9, 34]. The default mode network includes the posterior cingulate/retrosplenial cortex, the bilateral inferior parietal cortex, the medial prefrontal cortex, the anterior cingulate cortex, and the medial/lateral temporal lobe [9, 17, 62]. The default mode network is active at rest and gets suppressed (deactivated) during successful memory formation and goal-directed behavior. Lu et al. demonstrated that olfactory deficits in the early stage of AD are accompanied by the disruption of the connectivity not only of the olfactory but also of the default mode network [97].

**Fig. 2:**
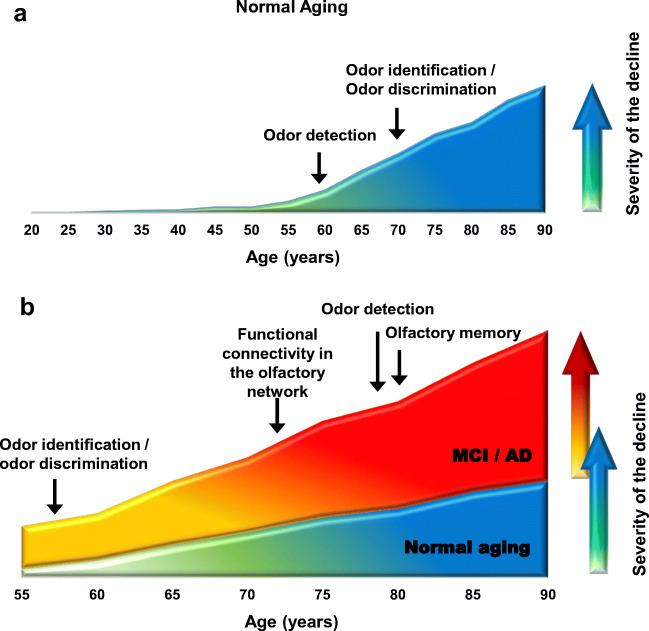
Summary of the known olfactory impairments during normal aging and the development of AD. **a** Human data showing at which age the decline in the given ability occurs, compared to young adult subjects. **b** Human data showing at which age the AD-related decline in the given ability occurs. Here, in contrast to panel **a**, NEC subjects serve as a reference. Because of the lack of reliable longitudinal studies of the mentioned capabilities, the arrows in the given graph reflect the age of the cohort under study rather than the true age when the given impairment appears. Data are taken from refs. [41, 43, 44, 68, 78, 96, 97, 117, 128, 143, 144]. *Y*-axis is shown in arbitrary units

Figure 2 summarizes the human data showing at which age the decline in the given ability occurs with aging, compared to young adult subjects (Fig. 2a), and with the development of AD, compared to normal elderly controls (Fig. 2b). As shown in Fig. 2b, in AD, all aspects of the odor sensing and processing of the odor information are impaired.

## AD-related olfactory deficits are recapitulated in mouse models of the disease

The olfactory system is phylogenetically conserved in a broad array of animals [1] with men and mice sharing striking similarities in the nature of odorant receptor proteins, molecular mechanisms underlying the transduction of olfactory signals, organization of the peripheral and central olfactory pathways, as well as odor-guided behavior and olfactory memory. Moreover, same odorants were reported to be similarly attractive to mice and humans, revealing a component of olfactory preference conserved across the two species [100]. Therefore, mice represent a valid model for analyzing molecular mechanisms underlying both aging- and AD-related changes in olfaction.

In patients with AD, the functional impairment of the OB occurs in the presence of neurofibrillary tangles and amyloid plaques, with plaque load increasing during disease progression [8]. Animal models of the disease, for brevity referred to as AD mice, also show co-occurrence of the olfactory dysfunction and amyloid plaque deposition. The AD mice usually overexpress amyloid precursor (APP), presenilin 1 (PS1), presenilin 2 (PS2), or tau proteins with mutations, known to cause a familiar form of AD in humans (see Table 1). Some recently established models are knockin mice, with known familiar mutations introduced in the respective mouse gen [136].

**Table 1:** Olfactory dysfunction in mouse models of AD

Number	Mouse model	Mutation	Histological hallmarks of AD in the OB	The nature of olfactory dysfunction	References
1	APP/PS1	APP swe (K595N/M596L)PS1dE9	Deposition of Aβ plaques in GCL as early as 4 months of age.	Impaired (i) odor detection, (ii) odor discrimination, (iii) olfactory memory	[119, 37, 177, 94, 130, 137, 164, 168]
				Impaired inter-bulbar functional connectivity (spontaneous and odor-evoked activities)	[94]
				Aβ-mediated deregulation of the OB proteome including genes involved in cytoskeletal stability and synaptic plasticity	[177]
			Decreased somatostatin, calretinin, and parvalbumin expression in the OB interneurons		[37, 137]
			Aβ is deposited in the olfactory nerve layer at 1–2 months of age, in the GL, EPL, and MCL at 3–4 months of age. At 8–9 months, all layers contain Aβ deposits.		[164, 168]
2	Tg2576	hAPP swe	Deposition of Aβ plaques in the GL and the GCL as early as 3–4 months of age	Impaired (i) habituation to repeated odor presentations, (ii) odor discrimination, (iii) olfactory memory	[161, 162, 171]
				Aberrant network function with increased spontaneous activity in the OB and the piriform cortex (PCX), heightened odor-evoked activity in the PCX, suppressed odor-evoked activity in the OB, increased OB–PCX functional connectivity	[162]
				Disrupted olfactory sensory neuron (OSN) axon-targeting, impaired innate olfactory behavior, reduced expression of activity-dependent genes in the OB due to selective overexpression of hAPP in OSNs.	[24]
3	3xTg	hAPP Swe PS1 M146V tau P301L	(i) Sparse Aβ deposition in the GCL at 20 months of age, (ii) decreased neurogenesis, (iii) DNA damage, (iv) apoptosis	Impaired olfactory memory	[25, 105]
4	Tα1-3RT mice	Overexpress human τ in neurons and glia	τ Protein appears in the EPL and the anterior olfactory nucleus at 6 months of age	Impaired odor detection	[178]
5	Tau P301S (PS19Tg)	MAPT P301S	Excessive P-tau expression in mitral cells as early as 1 month of age and throughout all layers of the OB including the GCL, MCL, and EPL at 5 months of age	Impaired (i) odor detection, (ii) odor discrimination, (iii) olfactory memory	[92, 179]
				Impairment of dendrodendritic synaptic structures between MC and GC from 5 months of age. Decrease in firing frequencies of mitral cells from 2 months on. A significant increase in the power spectra of gamma/low gamma oscillations at 9 months of age	[92]
				Impairment of gamma oscillatory activity and phase-amplitude theta-gamma coupling in the OB circuit starting at 3 months of age. Impairment of the short-term plasticity at 3 months of age, and long-term potentiation at 6 months of age	[4]
6	5xFAD mice (Tg6799)	APPSwFlLon, PSEN1*M146L*L286V 6799Vas	Deposition of Aβ plaques in the GCL at 6 months of age	Impaired (i) habituation to repeated odor presentations, (ii) odor discrimination, (iii) olfactory memory	[22, 61, 159]
7	ApoE KO mice	ApoE ^tm1Unc^		Impaired odor detection	[115, 175]
				Increased power of gamma oscillations in the OB at 3–5 months of age	[175]

In Tg2576 mice (Table 1), for example, Aβ first deposits in the OB almost a year before its deposition in the piriform and entorhinal cortex or hippocampus [161]. Inputs from the olfactory sensory neurons in the nose synapse on the dendrites of mitral and tufted cells in the glomerular layer (GL) of the OB, containing also the local interneurons (juxtaglomerular cells). Other OB layers include the external plexiform layer (EPL), mitral cell layer (MCL), and granule cell layer (GCL) [112]. In Tg2576 mice, Aβ deposition within particular layers of the OB is age-dependent, starting in the GL at 3–4 months of age and spreading to the GCL 10–12 months later [161]. The degree of amyloid deposition in these mice significantly correlates with the impairment of odor habituation as well as atypical odor discrimination and novel odor investigation behaviors [161]. Interestingly, the odor habituation behavior (see below) could be rescued by acute treatment with the liver-X receptor agonist GW3965, promoting proteolytic degradation of Aβ [162].

In the 3xTg mice (Table 1), sparse Aβ deposition in the GCL and a decreased neurogenesis in the OB were found by 20 months of age [106]. Likewise, the GCL was found to be preferentially affected by amyloid plaques in APP/PS1 mice (Table 1), which co-express human familiar mutations in APP and PS1 proteins [37, 94, 119, 130, 137, 168]. In addition, behavioral tests performed in middle-aged (9 and 12 months old) APP/PS1 mice revealed an impairment in odor detection, odor discrimination, and odor recognition memory as well as a significantly longer latency to find buried food pellets [130, 168].

### Odor detection

The buried food and the olfactory habituation/dishabituation tests are two common behavioral tests to analyze the animal’s olfaction. The buried food test is used to assess the ability to smell volatile odors by using olfactory cues (chow, cookies, or food pellets) and the natural ability for foraging. The olfactory habituation/dishabituation test is used to evaluate the ability to detect and distinguish known and novel odors. The APP/PS1 mice show a decline in odor detecting ability (revealed by the habituation/dishabituation test) as early as 3 months of age and cognitive dysfunction (revealed by the Morris water maze test assessing spatial learning and memory) at the age of 9–10 months [164, 168]. Another study, however, reported similar olfactory detection ability in the buried food test for 13-month-old APP/PS1 and WT mice [27]. For the 5xFAD mouse model (Table 1), the buried food test revealed a decline in the odor detection ability in 6-month-old males [165], but not females [79]. Females, however, showed an age-related reduction in interest for social odors and a reduced investigative behavior towards novel conspecifics [79].

Transgenic mice overexpressing mutated tau protein (both P301L and P301S strains; Table 1) also showed a significant impairment in the olfactory detection ability, increasing with advancing age [69, 92]. Taken together, the data clearly show that different mouse models of AD recapitulate the AD-related odor detection deficits seen in human patients.

### Odor discrimination

Odor discrimination requires the piriform cortex [10] and an intact function of the local OB circuits [52]. Both regions present with profound Aβ deposition in mouse models of AD [81, 86, 161, 164, 173]. The spontaneous odor discrimination ability in animals is evaluated with the help of the cross-habituation test [32]. This test relies on the animal’s instinctive curiosity and interest in novel stimuli. After a repeated presentation of the same odorant (aiming to cause habituation to this odorant), a novel odorant is introduced and the time spent investigating the novel odorant compared to the time spent investigating the known odorant is recorded.

The first study, using the cross-habituation test to evaluate the ability of odor discrimination, revealed lower odor discrimination abilities in Tg2576 compared to age-matched WT mice at 6–7, 16, and 21–29 months of age [161]. Similar dysfunction was found in 6-month-old P301L tau mice [69]. Additionally, Wesson et al. investigated the relationship between Aβ deposition and age-dependent deficits in odor discrimination. They found that specific behavioral impairments developed in line with a progressive Aβ burden in specific olfactory regions. For example, first non-fibrillar Aβ deposits within the GL of the OB appeared as early as 3 months of age but odor discrimination deficits appeared later, together with Aβ deposits within the piriform and entorhinal cortices [161].

### Olfactory memory

The olfactory learning and memory task relies on the animal’s ability to associate the olfactory-guided food-seeking with, for example, a water reward. The animals learn this connection by getting a reward after choosing the right odor. A task can be made more complex by introducing a so-called olfactory H-maze, in which the mice had to remember at which of the four ends of H they have already obtained their reward in order to succeed in either alternating or non-alternating learning task [39, 61, 156]. In 5xFAD mice, the olfactory H-maze task revealed olfactory memory impairment as early as 4 months of age [61], whereas olfactory memory deficits in the olfactory habituation test were seen at 6–8 months of age [159].

The social transmission of food preference task is used to evaluate olfactory memory without the spatial learning component. This task uses conspecifics as a cue to lead mice to choose their food [59, 60]. The 18-month-old 3xTg mice (Table 1) exhibited pronounced deficits in odor-based memory, as revealed by the social transmission of food preference task [25]. In Tg2576 mice, olfactory memory impairment developed already at 4 months of age and was getting increasingly more severe with aging [162, 171].

Taken together, many different strains of AD mice exhibit an impairment of olfactory memory, which is getting more severe as the disease progresses.

### Functional connectivity in the olfactory network

As already mentioned above, odor information enters the OB via the axons of the olfactory sensory neurons and is further processed by ON microcircuits containing mitral/tufted, granule, and juxtaglomerular cells [89]. This process is accompanied by gamma oscillations [121], including low (40–70 Hz) and high (70–100 Hz) gamma [88]. Besides gamma oscillations, phase-amplitude coupling was also reported to be involved in the processing of odor information [95]. Deficits in both OB gamma network oscillations and phase-amplitude coupling were found in aging and AD mice [2–4, 12]. P301S mice not only exhibit AD-related olfactory dysfunction and cognitive decline but also present with excessive tau hyperphosphorylation, particularly in mitral cells [92, 169]. Consistently, a significant decrease in firing frequencies of mitral cells (seen already at 2 months of age) and a significant increase in the power spectra of gamma and low gamma were observed in P301S mice [92]. A recent study showed that the P301S mice exhibit impairments in gamma oscillatory activity and phase-amplitude theta-gamma coupling (PAC) in the OB circuit, but not in the entorhinal cortex as well as hippocampal CA1 and CA3 regions, starting at 3 months of age. Besides, fEPSP recording showed a deficit in the short-term plasticity at 3 months of age, while both short-term plasticity and long-term potentiation showed deficits at 6 months of age [4].

In summary, P-tau might contribute to olfactory dysfunction in AD by decreasing firing frequencies of mitral cells, impairing the structure of synaptic contacts between MC and GC as well as PAC, and increasing the power spectra of gamma and low gamma in the OB. However, the role of Aβ accumulation for the functional connectivity of olfactory networks in AD still remains unclear.

## Common cellular/molecular mechanisms underlying impairment of olfaction

The observation that odor detection ability declines in the elderly population, late-stage AD patients and AD mice, enables the use of mouse models of AD for understanding the cellular/molecular mechanisms of this pathology.

One of the known molecular mechanisms is the accumulation of Aβ itself, as its accumulation in the olfactory system correlates with the olfactory impairment. Even in the absence of amyloid plaques, soluble Aβ triggered axonal dysfunction in the olfactory epithelium and impaired innate aversive or appetitive responses of mutant mice [24]. Consistently, APP/PS1 mice also showed impairment in the odor detection capability at 3–4 months, right after the non-fibrillar Aβ deposits were detected in the MCL [164]. In 5xFAD mice, however, the intracellular accumulation of soluble Aβ (clearly present in 3-month-old animals) was insufficient to impair learning and memory as well as odor discrimination abilities [165]. The latter became impaired only after the extracellular deposition of amyloid plaques in 6-month-old mice. The causal relationship between Aβ and the impairment of olfaction was obtained using the intrabulbar injection of soluble Aβ oligomers [6]. Two weeks after intrabulbar Aβ injection, experimental rats and mice showed a significant reduction in the odor detection ability (buried food test). This impairment was not related to alterations in motor performance or motivation to seek food and correlated with the formation of Aβ deposits with the bulb. By recording extracellular local field potentials from the GCL in OB slices, the same authors have shown that bath application of clinically relevant concentrations (30 nM) of Aβ oligomers for 1 h induces a significant, concentration-dependent, and reversible inhibition of the neural network activity in the OB [6]. In vivo, local field potential recordings in the OB of Tg2576 mice also revealed abnormal spontaneous network activity at just 3–4 months of age [162]. This time, however, Aβ deposition correlated with neural network hyperactivity, accompanied by prominent increases in both beta- and gamma-band power not only in the OB but also in the piriform cortex. This hyperactivity persisted until later in life when the network converted to a hypoactive state. This conversion was also Aβ-dependent, because liver-X receptor agonist treatment, which promotes Aβ degradation, was able to normalize the neural network activity as well as olfactory behavior [162]. These Aβ-mediated effects on the activity of neural networks are not unique to the olfactory system and were observed previously in the cortex as well as in the hippocampus [7, 19, 20, 66]. Moreover, AD patients as well as mouse models of AD are more susceptible to epileptic seizures than the respective controls [66, 122], and epilepsy is often accompanied by robust olfactory deficits, including deficits in odor detection, identification, discrimination, and olfactory memory [51, 75].

Accumulation of Aβ and formation of the amyloid plaques inevitably cause neuroinflammation and activation of the brain’s immune system [124]. Consistently, a significant increase in the density of amoeboid (i.e., activated and phagocytic) microglia, the innate immune cells of the brain, was observed within the anterior olfactory nucleus and the OB of AD patients [45]. In line with the ability of activated microglia to produce pro-inflammatory cytokines [31, 126, 132, 148], aged APP/PS1 mice exhibited a significant increase in expression of mRNA encoding for interleukin-1β, tumor necrosis factor-α, and chemokine MCP1 in the OB [129]. The levels of pro-inflammatory factors like monocyte chemoattractant protein-1 (MCP-1/CCL2) and interleukin-12 were also significantly higher in the olfactory mucus of elderly (65–80 years old) compared to young (21–40 years old) subjects [160]. Similar data were obtained in mice, with the expression of the mRNA of pro-inflammatory cytokine interleukin-6 in the nasal mucosa of aged (16-month-old) mice being significantly higher than that in 2-month-old mice [155].

Apolipoprotein E (ApoE) is another interesting molecule contributing to the impairment of olfaction in mice and humans. A lipid transport protein ApoE is expressed in the brain, where it serves as a cholesterol transporter, and is enriched in the olfactory brain structures [114, 116]. ApoE is polymorphic, with three major alleles (ɛ2, ɛ3, and ɛ4). The ApoE-ɛ4 represents a known genetic risk factor for the development of AD [93]. Interestingly, elderly (75–80 years old) ɛ4 carriers have a higher propensity to develop both olfactory impairment and a decline in episodic memory [120]. Olfactory dysfunction is also prevalent among obese elderly (60–90 years old) ɛ4 carriers [145]. Besides olfactory dysfunction, ApoE4 was shown to exacerbate AD-related pathology by (i) increasing β pathway processing of full-length APP and, therefore, Aβ production; (ii) decreasing astrocyte- and microglia-mediated Aβ clearance; (iii) reducing neuroprotective Sirtuin T1 expression; (iv) inducing mitochondrial dysfunction and lysosomal leakage; (v) triggering Tau and APP phosphorylation; (vi) inhibiting insulin signaling; and (vii) inducing programmed cell death [23, 153, 176].

The correlation between ApoE and olfactory impairment was also found in animal studies. Thus, ApoE^−/−^ mice exhibited olfactory deficits in the buried food test, indicating that ApoE deficiency or dysfunctionality may be related to olfaction deficits [115, 175]. Interestingly, in vivo local field potential recordings in the OB of 3–5-month-old ApoE^−/−^ mice revealed selective neural network hyperactivity in the gamma frequency range, accompanied by an increased number of adult-born and parvalbumin-expressing interneurons [175].

In humans, olfactory dysfunction is also significantly related to insulin resistance [104]. On the other hand, the impairment of the neuronal insulin signaling increases the concentration of toxic Aβ oligomers and hyperphosphorylation of tau protein [11, 76]. The CNS insulin resistance may promote AD by enhancing pathological phosphorylation of tau [76] and production of Aβ_42_, an aggregation-prone variant of Aβ [113]. In aging mice, downregulation of insulin-like growth factor production, a high level of which is related to impaired glucose tolerance and a higher risk of type 2 diabetes in humans [57], improves OB neurogenesis and olfactory memory [26]. In addition, treating 7-month-old APP/PS1 mice with antidiabetics liraglutide decreased amyloid plaque load and the level of soluble amyloid oligomers in the cortex, along with prevention of the synapse loss and deterioration of synaptic plasticity in the hippocampus, commonly observed in this mouse model of AD [103].

Because the degree of adult neurogenesis in the OB correlates with olfactory memory function [5, 16, 64, 99, 133, 155], the aging- or AD-related olfactory impairment might in part be caused by the impaired neurogenesis. Previous studies have shown that middle-aged (12-month-old) mice have a lower density of the subventricular zone–derived neuroblasts in the OB and a lower stem cell activity in the subventricular zone compared to young adult (2-month-old) mice [13]. Moreover, a decreased number of the olfactory receptor neurons were found in the olfactory epithelium of aged (16-month-old) mice [155], suggesting that age-related olfactory impairment might be caused by the depletion of neural stem cells, maintaining the population of the olfactory receptor neurons. In young control mice, the experimentally induced abolishment of the adult neurogenesis caused a drastic reduction in the short-term olfactory memory leaving, however, the odor discrimination ability and the long-term olfactory memory intact [16]. Both adult neurogenesis and short-term olfactory memory are also impaired in AD mice [30, 37, 64, 159, 161, 174]. In 6.5–8-month-old Tg2576 mice, for example, this reduction was likely caused by poor migration and survival of adult-born cells due to degeneration of the locus coeruleus and a reduced expression of the polysialylated neuronal cell adhesion molecule (PSA-NCAM) [64].

Taken together, these data reveal a plentitude of different, often interdependent, mechanisms contributing to aging- and AD-associated olfactory impairment in men and mice. The latter include the activation of the innate immune system and production of pro-inflammatory species, decreased trophic support, impairment of adult neurogenesis in the olfactory epithelium and the OB, and dysfunctional lipid and protein homeostasis. The sheer amount of modulatory pathways involved accounts for the enhanced sensitivity of the olfactory system and enables it to serve as an early biomarker of aging-associated damage or disease.

## References

[1] Ache BW, Young JM (2005). Olfaction: diverse species, conserved principles. Neuron.

[2] Ahnaou A, Moechars D, Raeymaekers L, Biermans R, Manyakov NV, Bottelbergs A, Wintmolders C, Van Kolen K, Van De Casteele T, Kemp JA, Drinkenburg WH (2017). Emergence of early alterations in network oscillations and functional connectivity in a tau seeding mouse model of Alzheimer’s disease pathology. Sci Rep.

[3] Ahnaou A, Walsh C, Manyakov NV, Youssef SA, Drinkenburg WH (2019). Early electrophysiological disintegration of hippocampal neural networks in a novel locus coeruleus tau-seeding mouse model of Alzheimer’s disease. Neural Plast.

[4] Ahnaou A, Rodriguez-Manrique D, Biermans R, Embrechts S, Manyakov NV, Drinkenburg WH (2020). Functional alterations in the olfactory neuronal circuit occur before hippocampal plasticity deficits in the P301S Mouse model of tauopathy: implications for early diagnosis and translational research in Alzheimer’s disease. Int J Mol Sci.

[5] Alonso M, Viollet C, Gabellec M-M, Meas-Yedid V, Olivo-Marin J-C, Lledo P-M (2006). Olfactory discrimination learning increases the survival of adult-born neurons in the olfactory bulb. J Neurosci.

[6] Alvarado-Martínez R, Salgado-Puga K, Peña-Ortega F (2013). Amyloid beta inhibits olfactory bulb activity and the ability to smell. PLoS One.

[7] Asavapanumas N, Brawek B, Martus P, Garaschuk O (2019). Role of intracellular Ca2+ stores for an impairment of visual processing in a mouse model of Alzheimer’s disease. Neurobiol Dis.

[8] Attems J, Lintner F, Jellinger KA (2005). Olfactory involvement in aging and Alzheimer’s disease: an autopsy study. J Alzheimer’s Dis.

[9] Banks SJ, Zhuang X, Bayram E, Bird C, Cordes D, Caldwell JZK, Cummings JL (2018). Default mode network lateralization and memory in healthy aging and Alzheimer’s disease. J Alzheimer’s Dis.

[10] Barnes DC, Hofacer RD, Zaman AR, Rennaker RL, Wilson DA (2008). Olfactory perceptual stability and discrimination. Nat Neurosci.

[11] Bedse G, Di Domenico F, Serviddio G, Cassano T (2015). Aberrant insulin signaling in Alzheimer’s disease: current knowledge. Front Neurosci.

[12] Booth CA, Ridler T, Murray TK, Ward MA, de Groot E, Goodfellow M, Phillips KG, Randall AD, Brown JT (2016). Electrical and network neuronal properties are preferentially disrupted in dorsal, but not ventral, medial entorhinal cortex in a mouse model of tauopathy. J Neurosci.

[13] Bouab M, Paliouras GN, Aumont A, Forest-Bérard K, Fernandes KJL (2011). Aging of the subventricular zone neural stem cell niche: evidence for quiescence-associated changes between early and mid-adulthood. Neuroscience.

[14] Braak H, Braak E (1997). Frequency of stages of Alzheimer-related lesions in different age categories. Neurobiol Aging.

[15] Braak H, Braak E (1997). Staging of Alzheimer-related cortical destruction. Int psychogeriatrics.

[16] Breton-Provencher V, Lemasson M, Peralta MR, Saghatelyan A (2009). Interneurons produced in adulthood are required for the normal functioning of the olfactory bulb network and for the execution of selected olfactory behaviors. J Neurosci.

[17] Buckner RL, Andrews-Hanna JR, Schacter DL (2008). The brain’s default network: anatomy, function, and relevance to disease. Ann N Y Acad Sci.

[18] Busche MA, Hyman BT (2020). Synergy between amyloid-β and tau in Alzheimer’s disease. Nat Neurosci.

[19] Busche MA, Eichhoff G, Adelsberger H, Abramowski D, Wiederhold K-H, Haass C, Staufenbiel M, Konnerth A, Garaschuk O (2008) Clusters of hyperactive neurons near amyloid plaques in a mouse model of Alzheimer’s disease. Science (80- ) 321:1686–1689. doi: 10.1126/science.1162844 10.1126/science.1162844 18802001

[20] Busche MA, Chen X, Henning HA, Reichwald J, Staufenbiel M, Sakmann B, Konnerth A (2012). Critical role of soluble amyloid- for early hippocampal hyperactivity in a mouse model of Alzheimer’s disease. Proc Natl Acad Sci.

[21] Caballero A, Reales JM, Ballesteros S (2018). Taste priming and cross-modal taste-olfactory priming in normal aging and in older adults with mild cognitive impairment. Psicothema.

[22] Cai Y, Xue ZQ, Zhang XM, Li MB, Wang H, Luo XG, Cai H, Yan XX (2012). An age-related axon terminal pathology around the first olfactory relay that involves amyloidogenic protein overexpression without plaque formation. Neuroscience.

[23] Campagna J, Spilman P, Jagodzinska B, Bai D, Hatami A, Zhu C, Bilousova T, Jun M, Elias CJ, Pham J, Cole G, LaDu MJ, Jung ME, Bredesen DE, John V (2018). A small molecule ApoE4-targeted therapeutic candidate that normalizes sirtuin 1 levels and improves cognition in an Alzheimer’s disease mouse model. Sci Rep.

[24] Cao L, Schrank BR, Rodriguez S, Benz EG, Moulia TW, Rickenbacher GT, Gomez AC, Levites Y, Edwards SR, Golde TE, Hyman BT, Barnea G, Albers MW (2012) Aβ 2 alters the connectivity of olfactory neurons in the absence of amyloid plaques in vivo. Nat Commun 3. 10.1038/ncomms2013 10.1038/ncomms2013 PMC3529477 22910355

[25] Cassano T, Romano A, Macheda T, Colangeli R, Cimmino CS, Petrella A, LaFerla FM, Cuomo V, Gaetani S (2011). Olfactory memory is impaired in a triple transgenic model of Alzheimer disease. Behav Brain Res.

[26] Chaker Z, Aïd S, Berry H, Holzenberger M (2015). Suppression of IGF-I signals in neural stem cells enhances neurogenesis and olfactory function during aging. Aging Cell.

[27] Cheng D, Logge W, Low JK, Garner B, Karl T (2013). Novel behavioural characteristics of the APPSwe/PS1ΔE9 transgenic mouse model of Alzheimer’s disease. Behav Brain Res.

[28] Cherry JA, Baum MJ (2020). Sex differences in main olfactory system pathways involved in psychosexual function. Genes, Brain Behav.

[29] Choudhury ES, Moberg P, Doty RL (2003). Influences of age and sex on a microencapsulated odor memory test. Chem Senses.

[30] Chuang TT (2010). Neurogenesis in mouse models of Alzheimer’s disease. Biochim Biophys Acta - Mol Basis Dis.

[31] Conti P, Lauritano D, Caraffa A, Gallenga CE, Kritas SK, Ronconi G, Martinotti S (2020). Microglia and mast cells generate proinflammatory cytokines in the brain and worsen inflammatory state: suppressor effect of IL-37. Eur J Pharmacol.

[32] Coronas-Samano G, Ivanova AV, Verhagen JV (2016). The habituation/cross-habituation test revisited: guidance from sniffing and video tracking. Neural Plast.

[33] Croy I, Zehner C, Larsson M, Zucco GM, Hummel T (2015). Test-retest reliability and validity of the sniffin’ TOM odor memory test. Chem Senses.

[34] Damoiseaux JS, Prater KE, Miller BL, Greicius MD (2012) Functional connectivity tracks clinical deterioration in Alzheimer’s disease. Neurobiol Aging 33:828.e19-828.e30. doi: 10.1016/j.neurobiolaging.2011.06.024 10.1016/j.neurobiolaging.2011.06.024 PMC3218226 21840627

[35] Davies DC, Brooks JW, Lewis DA (1993). Axonal loss from the olfactory tracts in Alzheimer’s disease. Neurobiol Aging.

[36] Dawson P, Rabold EM, Laws RL, Conners EE, Gharpure R, Yin S, Buono SA, Dasu T, Bhattacharyya S, Westergaard RP, Pray IW, Ye D, Nabity SA, Tate JE, Kirking HL (2020) Loss of taste and smell as distinguishing symptoms of coronavirus disease 2019. Clin Infect Dis 2008–2010. doi: 10.1093/cid/ciaa799 10.1093/cid/ciaa799 PMC7337666 32562541

[37] De la Rosa-Prieto C, Saiz-Sanchez D, Ubeda-Banon I, Flores-Cuadrado A, Martinez-Marcos A (2016). Neurogenesis, neurodegeneration, interneuron vulnerability, and amyloid-β in the olfactory bulb of APP/PS1 mouse model of Alzheimer’s disease. Front Neurosci.

[38] De Wijk RA, Cain WS (1994). Odor quality: discrimination versus free and cued identification. Percept Psychophys.

[39] Del’Guidice T, Nivet E, Escoffier G, Baril N, Caverni JP, Roman FS (2009). Perseveration related to frontal lesion in mice using the olfactory H-maze. Behav Brain Res.

[40] Devanand DP, Lee S, Manly J, Andrews H, Schupf N, Doty RL, Stern Y, Zahodne LB, Louis ED, Mayeux R (2015). Olfactory deficits predict cognitive decline and Alzheimer dementia in an urban community. Neurology.

[41] Dhilla Albers A, Asafu-Adjei J, Delaney MK, Kelly KE, Gomez-Isla T, Blacker D, Johnson KA, Sperling RA, Hyman BT, Betensky RA, Hastings L, Albers MW (2016). Episodic memory of odors stratifies Alzheimer biomarkers in normal elderly. Ann Neurol.

[42] Dintica CS, Marseglia A, Rizzuto D, Wang R, Seubert J, Arfanakis K, Bennett DA, Xu W (2019). Impaired olfaction is associated with cognitive decline and neurodegeneration in the brain. Neurology.

[43] Djordjevic J, Jones-Gotman M, De Sousa K, Chertkow H (2008). Olfaction in patients with mild cognitive impairment and Alzheimer’s disease. Neurobiol Aging.

[44] Doorduijn AS, de van der Schueren MAE, van de Rest O, de Leeuw FA, Fieldhouse JLP, Kester MI, Teunissen CE, Scheltens P, van der Flier WM, Visser M, Boesveldt S (2020). Olfactory and gustatory functioning and food preferences of patients with Alzheimer’s disease and mild cognitive impairment compared to controls: the NUDAD project. J Neurol.

[45] Doorn KJ, Goudriaan A, Blits-Huizinga C, Bol JGJM, Rozemuller AJ, Hoogland PVJM, Lucassen PJ, Drukarch B, Van De Berg WDJ, Van Dam AM (2014). Increased amoeboid microglial density in the olfactory bulb of Parkinson’s and Alzheimer’s Patients. Brain Pathol.

[46] Doty RL (2017). Olfactory dysfunction in neurodegenerative diseases: is there a common pathological substrate?. Lancet Neurol.

[47] Doty RL, Reyes PFGT (1987). Presence of both odor an detection deficits in Alzheimer disease. Brain Res Bull.

[48] Doty R, Shaman P, Applebaum S, Giberson R, Siksorski L, Rosenberg L (1984) Smell identification ability: changes with age. Science (80- ) 226:1441–1443. doi: 10.1126/science.6505700 10.1126/science.6505700 6505700

[49] Doty RL, Shaman P, Kimmelman CP, Dann MS (1984). University of pennsylvania smell identification test: a rapid quantitative olfactory function test for the clinic. Laryngoscope.

[50] Doty RL, Hawkes CH, Good KP, Duda JE (2015) Odor perception and neuropathology in neurodegenerative diseases and schizophrenia. Handb Olfaction Gustation Third Ed 403–452. doi: 10.1002/9781118971758.ch18

[51] Doty RL, Tourbier I, Neff JK, Silas J, Turetsky B, Moberg P, Kim T, Pluta J, French J, Sharan AD, Sperling MJ, Mirza N, Risser A, Baltuch G, Detre JA (2018). Influences of temporal lobe epilepsy and temporal lobe resection on olfaction. J Neurol.

[52] Doucette W, Milder J, Restrepo D (2007). Adrenergic modulation of olfactory bulb circuitry affects odor discrimination. Learn Mem.

[53] Eichenbaum H, Morton TH, Potter H, Corkin S (1983). Selective olfactory deficits in case H.M. Brain.

[54] Ferris CF, Stolberg T, Kulkarni P, Murugavel M, Blanchard R, Caroline CD, Febo M, Brevard M, Simon NG (2008). Imaging the neural circuitry and chemical control of aggressive motivation. BMC Neurosci.

[55] Fine LG, Riera CE (2019). Sense of smell as the central driver of Pavlovian appetite behavior in mammals. Front Physiol.

[56] Frank RA, Rybalsky K, Brearton M, Mannea E (2011). Odor recognition memory as a function of odor-naming performance. Chem Senses.

[57] Friedrich N, Thuesen B, Jrøgensen T, Juul A, Spielhagen C, Wallaschofksi H, Linneberg A (2012). The association between IGF-I and insulin resistance: a general population study in Danish adults. Diabetes Care.

[58] Fullard ME, Morley JF, Duda JE (2017). Olfactory dysfunction as an early biomarker in Parkinson’s disease. Neurosci Bull.

[59] Galef BG, Wigmore SW (1983). Transfer of information concerning distant foods: a laboratory investigation of the “information-centre” hypothesis. Anim Behav.

[60] Galef BG, Iliffe CP, Whiskin EE (1994). Social influences on rats’ (Rattus norvegicus) preferences for flavored foods, scented nest materials, and odors associated with harborage sites: are flavored food special?. J Comp Psychol.

[61] Girard SD, Baranger K, Gauthier C, Jacquet M, Bernard A, Escoffier G, Marchetti E, Khrestchatisky M, Rivera S, Roman FS (2013). Evidence for early cognitive impairment related to frontal cortex in the 5XFAD mouse model of Alzheimer’s disease. J Alzheimer’s Dis.

[62] Gottfried JA (2010). Central mechanisms of odour object perception. Nat Rev Neurosci.

[63] Gottfried JA, Dolan RJ (2003). The nose smells what the eye sees: crossmodal visual facilitation of human olfactory perception. Neuron.

[64] Guérin D, Sacquet J, Mandairon N, Jourdan F, Didier A (2009). Early locus coeruleus degeneration and olfactory dysfunctions in Tg2576 mice. Neurobiol Aging.

[65] Hebert LE, Weuve J, Scherr PA, Evans DA (2013). Alzheimer disease in the United States (2010-2050) estimated using the 2010 census. Neurology.

[66] Hermes M, Eichhoff G, Garaschuk O (2010). Intracellular calcium signalling in Alzheimer’s disease. J Cell Mol Med.

[67] Hickman RA, Faustin A, Wisniewski T (2016). Alzheimer disease and its growing epidemic: risk factors, biomarkers and the urgent need for therapeutics. Neurol Clin.

[68] Hori Y, Matsuda O, Ichikawa S (2015). Olfactory function in elderly people and patients with Alzheimer’s disease. Psychogeriatrics.

[69] Hu Y, Ding W, Zhu X, Chen R, Wang X (2016). Olfactory dysfunctions and decreased nitric oxide production in the brain of human P301L tau transgenic mice. Neurochem Res.

[70] Hummel T, Sekinger B, Wolf SR, Pauli E, Kobal G (1997). “Sniffin” sticks’. Olfactory performance assessed by the combined testing of odor identification, odor discrimination and olfactory threshold. Chem Senses.

[71] Josefsson M, Larsson M, Nordin S, Adolfsson R, Olofsson J (2017). APOE-ϵ4 effects on longitudinal decline in olfactory and non-olfactory cognitive abilities in middle-aged and old adults. Sci Rep.

[72] Jung HJ, Shin IS, Lee JE (2019). Olfactory function in mild cognitive impairment and Alzheimer’s disease: a meta-analysis. Laryngoscope.

[73] Karunanayaka P, Eslinger PJ, Wang JL, Weitekamp CW, Molitoris S, Gates KM, Molenaar PCM, Yang QX (2014). Networks involved in olfaction and their dynamics using independent component analysis and unified structural equation modeling. Hum Brain Mapp.

[74] Karunanayaka P, Tobia MJ, Yang QX (2017). Age-related resting-state functional connectivity in the olfactory and trigeminal networks. Neuroreport.

[75] Khurshid K, Crow AJD, Rupert PE, Minniti NL, Carswell MA, Mechanic-Hamilton DJ, Kamath V, Doty RL, Moberg PJ, Roalf DR (2019). A quantitative meta-analysis of olfactory dysfunction in epilepsy. Neuropsychol Rev.

[76] Kimura N (2016) Diabetes mellitus induces Alzheimer’s disease pathology: histopathological evidence from animal models. Int J Mol Sci 17. 10.3390/ijms17040503 10.3390/ijms17040503 PMC4848959 27058526

[77] Kitamura A, Torii K, Uneyama H, Niijima A (2010). Role played by afferent signals from olfactory, gustatory and gastrointestinal sensors in regulation of autonomic nerve activity. Biol Pharm Bull.

[78] Kondo K, Kikuta S, Ueha R, Suzukawa K, Yamasoba T (2020). Age-related olfactory dysfunction: epidemiology, pathophysiology, and clinical management. Front Aging Neurosci.

[79] Kosel F, Torres Munoz P, Yang JR, Wong AA, Franklin TB (2019). Age-related changes in social behaviours in the 5xFAD mouse model of Alzheimer’s disease. Behav Brain Res.

[80] Kotecha AM, Corrêa ADC, Fisher KM, Rushworth JV (2018). Olfactory dysfunction as a global biomarker for sniffing out Alzheimer’s disease: a meta-analysis. Biosensors.

[81] Kovács T (2004). Mechanisms of olfactory dysfunction in aging and neurodegenerative disorders. Ageing Res Rev.

[82] Kovács I, Török I, Zombori J, Yamaguchi H (1998). The olfactory bulb in Alzheimer’s disease. Acta Biol Hung.

[83] Kovács T, Cairns NJ, Lantos PL (1999). β-amyloid deposition and neurofibrillary tangle formation in the olfactory bulb in ageing and Alzheimer’s disease. Neuropathol Appl Neurobiol.

[84] Larsson M, Finkel D, Pedersen NL (2000). Odor identification: Influences of age, gender, cognition, and personality. Journals Gerontol - Ser B Psychol Sci Soc Sci.

[85] Larsson M, Hedner M, Papenberg G, Seubert J, Bäckman L, Laukka EJ (2016). Olfactory memory in the old and very old: relations to episodic and semantic memory and APOE genotype. Neurobiol Aging.

[86] Lehman EJH, Kulnane LS, Lamb BT (2003). Alterations in β-amyloid production and deposition in brain regions of two transgenic models. Neurobiol Aging.

[87] Leinwand SG, Chalasani SH (2011). Olfactory networks: from sensation to perception. Curr Opin Genet Dev.

[88] Lepousez G, Lledo PM (2013). Odor discrimination requires proper olfactory fast oscillations in awake mice. Neuron.

[89] Lepousez G, Valley MT, Lledo PM (2013). The impact of adult neurogenesis on olfactory bulb circuits and computations. Annu Rev Physiol.

[90] Li Q, Liberles SD (2015). Aversion and attraction through olfaction. Curr Biol.

[91] Li W, Howard JD, Gottfried JA (2010). Disruption of odour quality coding in piriform cortex mediates olfactory deficits in Alzheimer’s disease. Brain.

[92] Li S, Li W, Wu X, Li J, Yang J, Tu C, Ye X, Ling S (2019). Olfactory deficit is associated with mitral cell dysfunction in the olfactory bulb of P301S tau transgenic mice. Brain Res Bull.

[93] Liu C-C, Kanekiyo T, Xu H, Bu G (2013). Apolipoprotein E and Alzheimer disease: risk, mechanisms and therapy. Nat Rev Neurol.

[94] Liu Q, Li A, Gong L, Zhang L, Wu N, Xu F (2013). Decreased coherence between the two olfactory bulbs in Alzheimer’s disease model mice. Neurosci Lett.

[95] Losacco J, Ramirez-Gordillo D, Gilmer J, Restrepo D (2020). Learning improves decoding of odor identity with phase-referenced oscillations in the olfactory bulb. Elife.

[96] Lu J, Testa N, Jordan R, Elyan R, Kanekar S, Wang J, Eslinger P, Yang QX, Zhang B, Karunanayaka PR (2019) Functional connectivity between the resting-state olfactory network and the hippocampus in Alzheimer’s disease. Brain Sci 9. 10.3390/brainsci9120338 10.3390/brainsci9120338 PMC6955985 31775369

[97] Lu J, Yang QX, Zhang H, Eslinger PJ, Zhang X, Wu S, Zhang B, Zhu B, Karunanayaka PR (2019). Disruptions of the olfactory and default mode networks in Alzheimer’s disease. Brain Behav.

[98] Luers JC, Rokohl AC, Loreck N, Wawer Matos PA, Augustin M, Dewald F, Klein F, Lehmann C, Heindl LM (2020). Olfactory and gustatory dysfunction in coronavirus disease 19 (COVID-19). Clin Infect Dis.

[99] Mandairon N, Sacquet J, Garcia S, Ravel N, Jourdan F, Didier A (2006). Neurogenic correlates of an olfactory discrimination task in the adult olfactory bulb. Eur J Neurosci.

[100] Mandairon N, Poncelet J, Bensafi M, Didier A (2009). Humans and mice express similar olfactory preferences. PLoS One.

[101] Marin C, Vilas D, Langdon C, Alobid I, López-Chacón M, Haehner A, Hummel T, Mullol J (2018) Olfactory dysfunction in neurodegenerative diseases. Curr Allergy Asthma Rep 18. 10.1007/s11882-018-0796-4 10.1007/s11882-018-0796-4 29904888

[102] Mashta O (2007). Number of people in UK with dementia will more than double by 2050. BMJ.

[103] Mcclean PL, Parthsarathy V, Faivre E, Holscher C (2011). The diabetes drug liraglutide prevents degenerative processes in a mouse model of Alzheimer’s disease. J Neurosci.

[104] Min JY, Min KB (2018). Insulin resistance and the increased risk for smell dysfunction in US adults. Laryngoscope.

[105] Misiak MM, Hipolito MS, Ressom HW, Obisesan TO, Manaye KF, Nwulia EA (2017). Apo E4 alleles and impaired olfaction as predictors of Alzheimer’s disease. Clin Exp Psychol.

[106] Misiak M, Vergara Greeno R, Baptiste BA, Sykora P, Liu D, Cordonnier S, Fang EF, Croteau DL, Mattson MP, Bohr VA (2017). DNA polymerase β decrement triggers death of olfactory bulb cells and impairs olfaction in a mouse model of Alzheimer’s disease. Aging Cell.

[107] Mouly AM, Sullivan R (2009) Memory and plasticity in the olfactory system: from infancy to adulthood. Neurobiol Olfaction:367–413 21882422

[108] Muehlroth BE, Sander MC, Fandakova Y, Grandy TH, Rasch B, Lee Shing Y, Werkle-Bergner M (2020) Memory quality modulates the effect of aging on memory consolidation during sleep: reduced maintenance but intact gain. Neuroimage 209. doi: 10.1016/j.neuroimage.2019.116490 10.1016/j.neuroimage.2019.116490 PMC7068706 31883456

[109] Mundiñano IC, Caballero MC, Ordóñez C, Hernandez M, DiCaudo C, Marcilla I, Erro ME, Tuñon MT, Luquin MR (2011). Increased dopaminergic cells and protein aggregates in the olfactory bulb of patients with neurodegenerative disorders. Acta Neuropathol.

[110] Murphy C (1999). Loss of olfactory function in dementing disease. Physiol Behav.

[111] Murphy C, Cruickshanks KJ, Klein BEK, Klein R, Nondahl DM (2002). Prevalence of olfactory impairment. JAMA.

[112] Nagayama S, Homma R, Imamura F (2014). Neuronal organization of olfactory bulb circuits. Front Neural Circuits.

[113] Najem D, Bamji-Mirza M, Yang Z, Zhang W (2016). Aβ-induced insulin resistance and the effects of insulin on the cholesterol synthesis pathway and Aβ secretion in neural cells. Neurosci Bull.

[114] Nathan BP, Nisar R, Randall S, Short J, Sherrow M, Wong GK, Struble RG (2001). Apolipoprotein E is upregulated in olfactory bulb glia following peripheral receptor lesion in mice. Exp Neurol.

[115] Nathan BP, Yost J, Litherland MT, Struble RG, Switzer PV (2004). Olfactory function in apoE knockout mice. Behav Brain Res.

[116] Nathan BP, Nannapaneni S, Gairhe S, Nwosu I, Struble RG (2007). The distribution of apolipoprotein E in mouse olfactory epithelium. Brain Res.

[117] Naudin M, Mondon K, El-Hage W, Desmidt T, Jaafari N, Belzung C, Gaillard P, Hommet C, Atanasova B (2014). Long-term odor recognition memory in unipolar major depression and Alzheimer’s disease. Psychiatry Res.

[118] Ohm TG, Braak H (1987). Olfactory bulb changes in Alzheimer’s disease. Acta Neuropathol.

[119] Oi B, Lee S, Dickson T, Mitew S, Vickers J, Chuah M (2013). Denervation of the olfactory bulb leads to decreased Aβ plaque load in a transgenic mouse model of Alzheimer’ s disease. Curr Alzheimer Res.

[120] Olofsson JK, Nordin S, Wiens S, Hedner M, Nilsson L-G, Larsson M (2010). Odor identification impairment in carriers of ApoE-ɛ4 is independent of clinical dementia. Neurobiol Aging.

[121] Osinski BL, Kay LM (2016). Granule cell excitability regulates gamma and beta oscillations in a model of the olfactory bulb dendrodendritic microcircuit. J Neurophysiol.

[122] Palop JJ, Mucke L (2016). Network abnormalities and interneuron dysfunction in Alzheimer disease. Nat Rev Neurosci.

[123] Park SJ, Lee JE, Lee KS, Kim JS (2018). Comparison of odor identification among amnestic and non-amnestic mild cognitive impairment, subjective cognitive decline, and early Alzheimer’s dementia. Neurol Sci.

[124] Perry VH, Nicoll JAR, Holmes C (2010). Microglia in neurodegenerative disease. Nat Rev Neurol.

[125] Rahayel S, Frasnelli J, Joubert S (2012). The effect of Alzheimer’s disease and Parkinson’s disease on olfaction: a meta-analysis. Behav Brain Res.

[126] Ramirez AI, de Hoz R, Salobrar-Garcia E, Salazar JJ, Rojas B, Ajoy D, López-Cuenca I, Rojas P, Triviño A, Ramírez JM (2017). The role of microglia in retinal neurodegeneration: Alzheimer’s disease, Parkinson, and glaucoma. Front Aging Neurosci.

[127] Raviv JR, Kern RC (2004). Chronic sinusitis and olfactory dysfunction. Otolaryngol Clin North Am.

[128] Rawson NE, Gomez G, Cowart BJ, Kriete A, Pribitkin E, Restrepo D (2012). Age-associated loss of selectivity in human olfactory sensory neurons. Neurobiol Aging.

[129] Reale M, D’Angelo C, Costantini E, Di Nicola M, Yarla NS, Kamal MA, Salvador N, Perry G (2018). Expression profiling of cytokine, cholinergic markers, and amyloid-β deposition in the APP SWE/PS1dE9 mouse model of Alzheimer’s disease pathology. J Alzheimer’s Dis.

[130] Rey NL, Jardanhazi-Kurutz D, Terwel D, Kummer MP, Jourdan F, Didier A, Heneka MT (2012) Locus coeruleus degeneration exacerbates olfactory deficits in APP/PS1 transgenic mice. Neurobiol Aging 33:426.e1-426.e11. doi: 10.1016/j.neurobiolaging.2010.10.009 10.1016/j.neurobiolaging.2010.10.009 21109328

[131] Richardson JTE, Zucco GM (1989). Cognition and olfaction: a review. Psychol Bull.

[132] Riester K, Brawek B, Savitska D, Fröhlich N, Zirdum E, Mojtahedi N, Heneka MT, Garaschuk O (2020). In vivo characterization of functional states of cortical microglia during peripheral inflammation. Brain Behav Immun.

[133] Rochefort C, Gheusi G, Vincent J-D, Lledo P-M (2002) Enriched odor exposure increases the number of newborn neurons in the adult olfactory bulb and improves odor memory. J Neurosci 22:2679–89. doi: 20026260 10.1523/JNEUROSCI.22-07-02679.2002 PMC6758329 11923433

[134] Rolls ET (2019) Taste and smell processing in the brain, 1st ed. Elsevier B.V. 10.1016/B978-0-444-63855-7.00007-1 31604566

[135] Rumeau C, Nguyen DT, Jankowski R (2016). How to assess olfactory performance with the Sniffin’ Sticks test®. Eur Ann Otorhinolaryngol Head Neck Dis.

[136] Saito T, Matsuba Y, Mihira N, Takano J, Nilsson P, Itohara S, Iwata N, Saido TC (2014). Single app knock-in mouse models of Alzheimer’s disease. Nat Neurosci.

[137] Saiz-Sanchez D, De La Rosa-Prieto C, Ubeda-Bañon I, Martinez-Marcos A (2013). Interneurons and beta-amyloid in the olfactory bulb, anterior olfactory nucleus and olfactory tubercle in appxps1 transgenic mice model of Alzheimer’s disease. Anat Rec.

[138] Sarafoleanu C, Mella C, Georgescu M, Perederco C (2009). The importance of the olfactory sense in the human behavior and evolution. J Med Life.

[139] Savic I, Gulyas B, Larsson M, Roland P (2000). Olfactory functions are mediated by parallel and hierarchical processing. Neuron.

[140] Schab FR (1991). Odor memory: taking stock. Psychol Bull.

[141] Schemper T, Voss S, Cain WS (1981). Odor identification in young and elderly persons: sensory and cognitive limitations. Journals Gerontol.

[142] Schubert CR, Cruickshanks KJ, Klein BEK, Klein R, Nondahl DM (2011). Olfactory impairment in older adults: five-year incidence and risk factors. Laryngoscope.

[143] Schubert CR, Cruickshanks KJ, Fischer ME, Huang GH, Klein BEK, Klein R, Pankow JS, Nondahl DM (2012). Olfactory impairment in an adult population: the beaver dam offspring study. Chem Senses.

[144] Schubert CR, Fischer ME, Pinto AA, Klein BEK, Klein R, Cruickshanks KJ (2017). Odor detection thresholds in a population of older adults. Laryngoscope.

[145] Seubert J, Laukka EJ, Rizzuto D, Hummel T, Fratiglioni L, Bäckman L, Larsson M (2017). Prevalence and correlates of olfactory dysfunction in old age: a population-based study. Journals Gerontol - Ser A Biol Sci Med Sci.

[146] Seubert J, Kalpouzos G, Larsson M, Hummel T, Bäckman L, Laukka EJ (2020). Temporolimbic cortical volume is associated with semantic odor memory performance in aging. Neuroimage.

[147] Ship JA, Pearson JD, Cruise LJ, Brant LJ, Metter EJ (1996). Longitudinal changes in smell identification. Journals Gerontol - Ser A Biol Sci Med Sci.

[148] Smith JA, Das A, Ray SK, Banik NL (2012). Role of pro-inflammatory cytokines released from microglia in neurodegenerative diseases. Brain Res Bull.

[149] Stevenson RJ (2010). An initial evaluation of the functions of human olfaction. Chem Senses.

[150] Talamo BR, Rudel RA, Kosik KS, Lee VMY, Neff S, Adelman L, Kauer JS (1989). Pathological changes in olfactory neurons in patients with Alzheimer’s disease. Nature.

[151] Taylor JE, Lau H, Seymour B, Nakae A, Sumioka H, Kawato M, Koizumi A (2020). An evolutionarily threat-relevant odor strengthens human fear memory. Front Neurosci.

[152] ter Laak HJ, Renkawek K, Van Workum FPA (1994). The olfactory bulb in Alzheimer disease: a morphologic study of neuron loss, tangles, and senile plaques in relation to olfaction. Alzheimer Dis. Assoc. Disord..

[153] Theendakara V, Patent A, Libeu CAP, Philpot B, Flores S, Descamps O, Poksay KS, Zhang Q, Cailing G, Hart M, John V, Rao RV, Bredesen DE (2013). Neuroprotective sirtuin ratio reversed by ApoE4. Proc Natl Acad Sci U S A.

[154] Tobia MJ, Yang QX, Karunanayaka P (2016). Intrinsic intranasal chemosensory brain networks shown by resting-state functional MRI. Neuroreport.

[155] Ueha R, Shichino S, Ueha S, Kondo K, Kikuta S, Nishijima H, Matsushima K, Yamasoba T (2018). Reduction of proliferating olfactory cells and low expression of extracellular matrix genes are hallmarks of the aged olfactory mucosa. Front Aging Neurosci.

[156] Verin M, Partiot A, Pillon B, Malapani C, Agid Y, Dubois B (1993). Delayed response tasks and prefrontal lesions in man-evidence for self generated patterns of behaviour with poor environmental modulation. Neuropsychologia.

[157] Walliczek-Dworschak U, Pellegrino R, Taube F, Mueller CA, Stuck BA, Dworschak P, Güldner C, Steinbach S (2018). Chemosensory function before and after multimodal treatment in chronic rhinosinusitis patients. Laryngoscope.

[158] Wang J, Eslinger PJ, Smith MB, Yang QX (2005). Functional magnetic resonance imaging study of human olfaction and normal aging. Journals Gerontol Ser A Biol Sci Med Sci.

[159] Wang Y, Wu Z, Bai YT, Wu GY, Chen G (2017). Gad67 haploinsufficiency reduces amyloid pathology and rescues olfactory memory deficits in a mouse model of Alzheimer’s disease. Mol Neurodegener.

[160] Wang H, Jaen C, Yoshikawa K, Haneoka M, Saito N, Nakamura J, Adappa N, Cohen N, Dalton P (2018) Cytokine profile in human olfactory cleft mucus and associated changes in olfactory function. bioRxiv 332395. doi: 10.1101/332395 10.1038/s41598-018-35102-2 PMC6249231 30464187

[161] Wesson DW, Levy E, Nixon RA, Wilson DA (2010). Olfactory dysfunction correlates with amyloid-βburden in an Alzheimer’s disease mouse model. J Neurosci.

[162] Wesson DW, Borkowski AH, Landreth GE, Nixon RA, Levy E, Wilson DA (2011). Sensory network dysfunction, behavioral impairments, and their reversibility in an Alzheimer’s β-amyloidosis mouse model. J Neurosci.

[163] White TL, Møller P, Köster EP, Eichenbaum H, Linster C (2015) Olfactory memory. Handb Olfaction Gustation Third Ed 337–352. doi: 10.1002/9781118971758.ch15

[164] Wu N, Rao X, Gao Y, Wang J, Xu F (2013). Amyloid-β deposition and olfactory dysfunction in an alzheimer’s disease model. J Alzheimer’s Dis.

[165] Xiao NA, Zhang J, Zhou M, Wei Z, Wu XL, Dai XM, Zhu YG, Chen XC (2015). Reduction of glucose metabolism in olfactory bulb is an earlier Alzheimer’s disease-related biomarker in 5XFAD mice. Chin Med J (Engl).

[166] Xie W, Peng CK, Shen J, Lin CP, Tsai SJ, Wang S, Chu Q, Yang AC (2020). Age-related changes in the association of resting-state fMRI signal variability and global functional connectivity in non-demented healthy people. Psychiatry Res.

[167] Xue J, Guo H, Gao Y, Wang X, Cui H, Chen Z, Wang B, Xiang J (2019). Altered directed functional connectivity of the hippocampus in mild cognitive impairment and Alzheimer’s disease: a resting-state fMRI study. Front Aging Neurosci.

[168] Yao ZG, Hua F, Zhang HZ, Li YY, Qin YJ (2017). Olfactory dysfunction in the APP/PS1 transgenic mouse model of Alzheimer’s disease: morphological evaluations from the nose to the brain. Neuropathology.

[169] Yoshiyama Y, Higuchi M, Zhang B, Huang SM, Iwata N, Saido TCC, Maeda J, Suhara T, Trojanowski JQ, Lee VMY (2007). Synapse loss and microglial activation precede tangles in a P301S tauopathy mouse model. Neuron.

[170] Young JM, Trask BJ (2002). The sense of smell: genomics of vertebrate odorant receptors. Hum Mol Genet.

[171] Young JW, Sharkey J, Finlayson K (2009). Progressive impairment in olfactory working memory in a mouse model of mild cognitive impairment. Neurobiol Aging.

[172] Yu Q, Guo P, Li D, Zuo L, Lian T, Yu S, Hu Y, Liu L, Jin Z, Wang R, Piao Y, Li L, Wang X, Zhang W (2018). Olfactory dysfunction and its relationship with clinical symptoms of Alzheimer disease. Aging Dis.

[173] Zelaya MV, Pérez-Valderrama E, De Morentin XM, Tuñon T, Ferrer I, Luquin MR, Fernandez-Irigoyen J, Santamaría E (2015) Olfactory bulb proteome dynamics during the progression of sporadic Alzheimer’s disease: identification of common and distinct olfactory targets across Alzheimer-related co-pathologies. Oncotarget 6:39437–39456. doi: 10.18632/oncotarget.6254 10.18632/oncotarget.6254 PMC4741837 26517091

[174] Zhang C, McNeil E, Dressler L, Siman R (2007). Long-lasting impairment in hippocampal neurogenesis associated with amyloid deposition in a knock-in mouse model of familial Alzheimer’s disease. Exp Neurol.

[175] Zhang J, Hao C, Jiang J, Feng Y, Chen X, Zheng Y, Liu J, Zhang Z, Long C, Yang L (2018). The mechanisms underlying olfactory deficits in apolipoprotein E–deficient mice: focus on olfactory epithelium and olfactory bulb. Neurobiol Aging.

[176] Zhao N, Liu C-C, Van Ingelgom AJ, Martens YA, Linares C, Knight JA, Painter MM, Sullivan PM, Bu G (2017) Apolipoprotein E4 impairs neuronal insulin signaling by trapping insulin receptor in the endosomes. Neuron 96:115-129.e5. doi: 10.1016/j.neuron.2017.09.003 10.1016/j.neuron.2017.09.003 PMC5621659 28957663

[177] Mercedes Lachén-Montes, Andrea González-Morales, Xabier Martínez de Morentin, Estela Pérez-Valderrama, Karina Ausín, María Victoria Zelaya, Antonio Serna, Ester Aso, Isidro Ferrer, Joaquín Fernández-Irigoyen, Enrique Santamaría, (2016) An early dysregulation of FAK and MEK/ERK signaling pathways precedes the β-amyloid deposition in the olfactory bulb of APP/PS1 mouse model of Alzheimer's disease. Journal of Proteomics 148:149–158 10.1016/j.jprot.2016.07.032 27498392

[178] Jonathan B. Macknin, Makoto Higuchi, Virginia M.-Y. Lee, John Q. Trojanowski, Richard L. Doty, (2004) Olfactory dysfunction occurs in transgenic mice overexpressing human τ protein. Brain Research 1000 (1-2):174–178 10.1016/j.brainres.2004.01.047 15053964

[179] Sujeong Yang, Wei-Li Kuan, Maria Grazia Spillantini, Yoland Smith, (2016) Progressive tauopathy in P301S tau transgenic mice is associated with a functional deficit of the olfactory system. European Journal of Neuroscience 44 (6):2396–2403 10.1111/ejn.13333 27422327

